# Minimal-access video-assisted retroperitoneal and/or transperitoneal debridement (VARTD) in the management of infected walled-off pancreatic necrosis with deep extension: initial experience from a prospective single-arm study

**DOI:** 10.1186/s40001-023-01030-9

**Published:** 2023-02-09

**Authors:** Wanjie Wei, Yongliang Tang, Zuxiang Peng, Jun Xie, Zhaoxia Deng, Tao Yuan, Chun Tang, Ruxian Pi, Shunan Wang, Siqi Zhao, Lu Wang, Chunxue Li, Yaoli Wang, Peng Zhang, Zhengbin Wu, Yafeng Wan, Yan Ma, Wen Tang, Xianchun Liang, Kun Liu, Wei Wang, Xianyi Liang, Dongmei Zeng, Shan Li, Hongming Liu

**Affiliations:** 1 Department of Hepatobiliary Surgery, Daping Hospital, Army Medical University, 10 Changjiang Branch Rd, Yuzhong District, Chongqing, 400042 China; 2 Department of Emergency, Southwest Hospital, Army Medical University, Chongqing, China; 3 Department of Radiology, Daping Hospital, Army Medical University, Chongqing, China; 4 Department of General Surgery, Daping Hospital, Army Medical University, Chongqing, China; 5 Intensive Care Unit, Daping Hospital, Army Medical University, Chongqing, China; 6 Department of Gastroenterology, Daping Hospital, Army Medical University, Chongqing, China; 7 Department of Information, Daping Hospital, Army Medical University, Chongqing, China

**Keywords:** Necrotizing pancreatitis, Walled-off pancreatic necrosis, Extensive necrosis, Minimal-access debridement

## Abstract

**Background:**

The currently preferred minimally invasive approaches have substantially improved outcomes of infected walled-off pancreatic necrosis (iWON). However, iWON with deep extension (iWONde) still poses a tricky challenge for sufficient necrosis evacuation by one stand-alone approach, often requiring repeated interventions. The aim of this study was to assess the effectiveness and safety of a minimal-access video-assisted retroperitoneal and/or transperitoneal debridement (hereafter called VARTD) in the management of iWONde.

**Methods:**

Patients who had developed an iWONde were recruited to receive the VARTD in this prospective single-arm study. The primary efficacy endpoint was clinical improvement up to day 28 after the VARTD, defined as *a* ≥ 75% reduction in size of necrotic collection (in any axis) on CT and clinical resolution of sepsis or organ dysfunction. The primary safety endpoint was a composite of major complications or death during follow-up. Six-month postdischarge follow-up was available.

**Results:**

Between July 18, 2018, and November 12, 2020, we screened 95 patients with necrotizing pancreatitis; of these, 21 iWONde patients (mean [SD] age, 42.9 [11.7] years; 10 [48%] women) were finally enrolled. The primary efficacy endpoint was achieved by most participants (14/21, 67%). No participants required repeated interventions. The primary safety endpoint occurred in six patients (29%). Except one in-hospital death attributable to repeated intra-abdominal hemorrhage, others were discharged without any major complication.

**Conclusions:**

The VARTD approach appears to have a reasonable efficacy with acceptable complication rates and thus might be an option for improving clinical management of iWONde.

**Trial registration:**

This study is registered with Chinese Clinical Trial Registry (chictr.org.cn number, ChiCTR1800016950).

**Supplementary Information:**

The online version contains supplementary material available at 10.1186/s40001-023-01030-9.

## Introduction

Acute pancreatitis is one of the leading causes of gastrointestinal-related admission to hospital [1, 2]. Approximately, 10–20% of patients develop necrotizing pancreatitis (NP), which is associated with high mortality rates of 20–40% [1–4]. In the case of NP, necrosis of pancreatic parenchyma and/or peripancreatic tissues is categorized into two conditions according to the disease course demarcated by 4 weeks following the NP onset—acute necrotic collections (ANCs) and walled-off necrosis (WON) [1, 5–8]. The latter poses a prolonged and complicated clinical course [6–10]. Especially, when an infection occurs in the necrotic bed (i.e., infected WON, hereafter called iWON), it is strongly recommended that invasive interventions be used to perform a drainage, debridement, or necrosectomy of the necrotic collection [2, 3, 7, 9–12].

The invasive approaches for managing WON have evolved over the past decade [1–3]. Historically, open surgical debridement/necrosectomy was the mainstay of therapy [2, 13]. However, such open approach is associated with an increased composite endpoint of death or severe complications [4, 14–16]. At present, a “step-up” approach has been advocated to be favored over open surgical approach to combat the iWON [1–4, 7, 11, 14–20]. This sequentially applies percutaneous catheter drainage (PCD), alone or in combination with other minimal-access interventions, including endoscopic transluminal debridement/necrosectomy (ETD/ETN), video-assisted retroperitoneal debridement (VARD), and sinus tract endoscopy (STE) [1–3, 7, 9, 11, 16–23].

Nevertheless, in the case of iWON with deep extension (iWONde, i.e., the necrosis is diffusely distributed throughout the abdomen), it is something that still represents a challenging scenario for those minimal-access approaches [2, 3]. In this instance, debridement of the iWONde in many cases requires repeated interventions (percutaneous and/or endoscopic) or even additional open necrosectomy if needed because of the difficulty in achieving sufficient evacuation of the large burden and deep extension of the necrotic collections [2, 3, 10]. For example, the endoscopic step-up approaches are capable of debriding the necrosis located along the peri-gastric or duodenal regions; however, this fails to access the necrosum in the setting of the necrosis extends into areas that are distant from stomach [3]. Another example is that when the necrosis extends to the right of the mesenteric vessels, and it is considered to be refractory to the VARD approach [2]. Thus, some NP patients who had developed an iWONde, in the current era, still necessarily undergo open necrosectomy, which is deemed the best alternative on this occasion in spite of its related high mortality rates [1–3, 10, 12, 24–27].

In this study, we attempted to solve the dilemma that no stand-alone minimal-access approach existed is suited for management of the infected necrosum with deep extension after failure of the “step-up” approach. Here, we introduced a minimal-access video-assisted retroperitoneal and/or transperitoneal debridement (hereafter called VARTD) that applies multi-mini-incision access for providing a practicable avenue to achieve sufficient clearance of the large avascular necrosis as much as possible, along with a continuous postoperative lavage by way of the flow that flushes from upper cavity towards lower zone, which may allow easy to irrigation and drainage of residual necrotic debris. The aim of this prospective single-arm study was to assess the effectiveness and safety of the VARTD for the iWONde.

## Methods

### Design, setting and participants

**Fig. 1:**
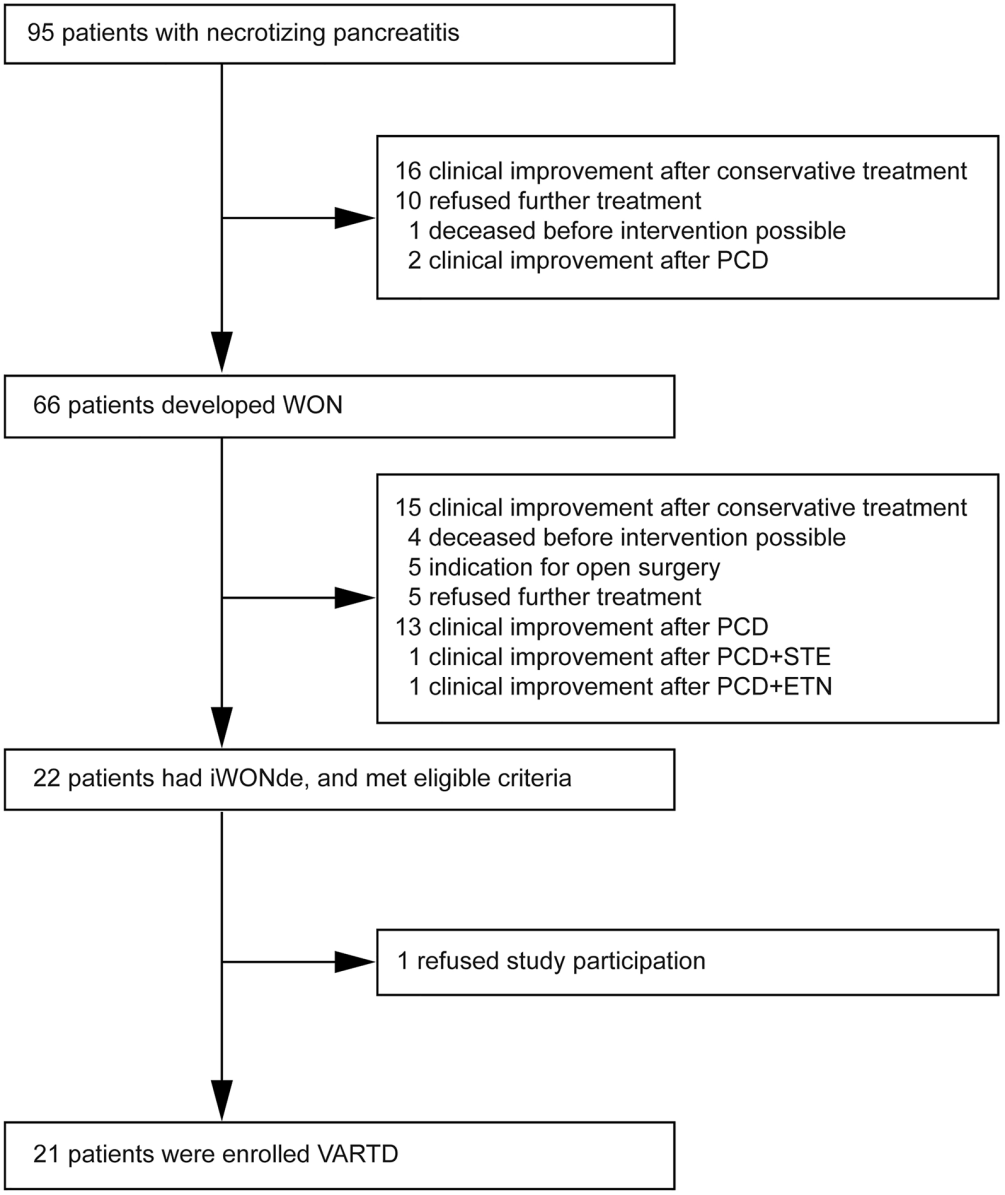
Patient flow diagram. WON, walled-off necrosis; iWONde, infected walled-off necrosis with deep extension; PCD, percutaneous catheter drainage; STE, sinus tract endoscopy; ETN, endoscopic transluminal necrosectomy; VARTD, video-assisted retroperitoneal and/or transperitoneal debridement

Patients admitted in our high-volume pancreas center with a diagnosis of necrotizing pancreatitis were screened for enrollment (Fig. 1). The inclusion criteria were (1) a diagnosis of WON was made by contrast-enhanced computed tomography (CECT); (2) infection of necrotic collections was laboratory-confirmed (i.e., a positive culture of the necrotic collections) or clinically suspected (e.g., gas configuration in necrotic collections on the CECT imaging, persistence of sepsis-associated clinical signs, or progressive deterioration of clinical conditions), without evidence of other causes of infection [4, 28]; (3) WON with deep extension, and the necrosis collection at least extends to the right of the mesenteric vessels. Exclusion criteria consisted of inability to obtain informed consent (i.e., refused participation or further treatment), history of previous surgical or endoscopic drainage/necrosectomy, pancreatitis following trauma or surgery, chronic pancreatitis, or pregnancy [4, 28]. The enrolled patients were followed up every 3 months via telephone or conventional outpatient clinic appointments up to 6 months after discharge. All the authors had access to the study data and reviewed and approved the manuscript.

This single-center prospective single-arm trial was conducted at Daping Hospital, Army Medical University, China. This trial is registered with the Chinese Clinical Trial Registry, number ChiCTR1800016950. Ethical approval was obtained from the Ethics Board of Daping Hospital, Army Medical University, China (reference number: 2018-17). Signed informed consent was obtained from all participants or their legal representatives before enrollment.

### Technique of the VARTD approach

To ensure that all the participants undergo a more standardized and uniform VARTD approach to all the procedures and avoid biases caused by differences in surgical technical skill, allowing for evidence-based recommendations for its future use, the VARTD was performed by a team consisting of two experienced pancreatic surgeons (Y Tang and H Liu).

**Fig. 2:**
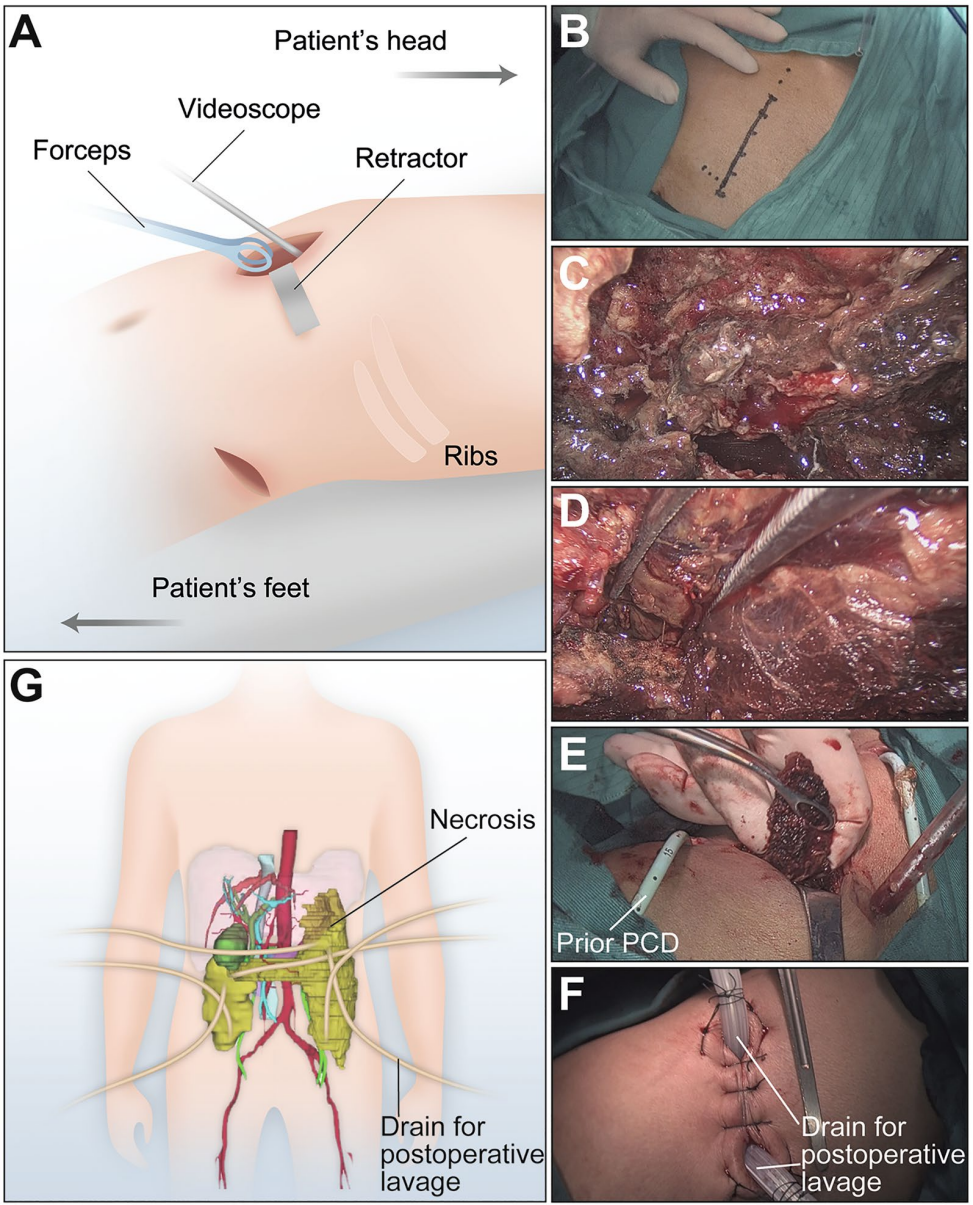
Technique of the VARTD approach. **A** Schematic diagram of the VARTD technique. Videoscope is inserted through the index approaches (i.e., epigastric, left and/or right retroperitoneal incisions). **B**–**F** Representative images obtained during the VARTD procedures. **B** Marking for incision used in planning of a retroperitoneal access. **C** A videoscope is inserted into the necrosis cavity, and residual necrotic debris is shown following fluid necrotic component is irrigated and aspirated. **D**, **E** Residual necrotic debris is removed with a sponge holding forceps under visualization. PCD, percutaneous catheter drainage. **F** Two large-bore tubes are crosswire placed in the necrosis cavity for postoperative drainage and lavage, followed by the necrosis cavity is closed and the incision is sutured. **G** Schematic diagram of location of drain placement. A representative 3D abdominal image (reconstructed from CT images) of a patient with infected walled-off pancreatic necrosis is presented (the necrosis is shown in yellow color)

After induction of general anesthesia, the patient was placed in the left/right lateral decubitus position. The decision about where to perform the skin incision depends on prior confirmation of the location of the necrosis on the CECT image, allowing the closest access route to the necrotic collection. First, a longitudinal/oblique mini-incision (approximately 3–5 cm) was made in the midaxillary line between the costal margin and the iliac crest (Fig. 2A, B). The prior PCD tubes serve as tracks, and exploratory puncture using 22-gauge needle was performed as an adjunct to determine an avascular access to the necrosis cavity. Once the wall of the necrosis cavity was opened by an electric cautery, a 10-mm, 30º camera, laparoscope was inserted into the necrotic cavity (Fig. 2A, C). Under visualization, fluid necrotic component was irrigated and aspirated with an 8-mm blunt suction cannula, and semi-solid necrotic mass was removed using a sponge holding forceps (Fig. 2A, D, E). Further, the necrotic material was irrigated with a solution of 3% hydrogen peroxide followed by 0.9% saline solution and suction. Two 24 Fr single-lumen silicone tubes were then placed into the necrosis cavity after achieving adequate hemostasis, which were interlaced with each other and positioned alongside the pancreatic tail and fossa iliaca, wherein one serves as an inflow port and the other as an outflow port for postoperative lavage (Fig. 2F, G). The two tubes were come straight out of the skin incisions. The necrosis cavity was closed with 3–0 Prolene running sutures, and the incision was sutured in layers. Subsequently, the patient was placed in the supine position, and a transverse upper midline incision or left rectus incision (approximately 6–8 cm) was made in the epigastric region. The gastrocolic ligament was divided near the greater curvature in an avascular plane to enter the lesser sac and reach the centrally located necrosis. The centrally located necrosis was debrided using the same technique. Two 24 Fr single-lumen silicone tubes were crosswire placed through the head and tail of the pancreas on the necrotic bed for postoperative lavage (Fig. 2F, G). The two tubes were brought out through the skin next to the epigastric incision. The centrally located and flank necrotic cavities were linked up with each other by blunt dissection with the surgeon’s fingers during operation. A planned feeding jejunostomy was carried out in patients who had severe gastrointestinal complaints, such as vomiting and bloating after meals. Continuous postoperative lavage with 0.9% saline solution (1–3 L per day) via the inflow to outflow tubes should be started early within 24–48 h post-operation.

### Clinical outcomes

To assess the efficacy of the combined VARTD and continuous postoperative lavage in the treatment of iWONde, we prespecified the primary efficacy endpoint as clinical improvement up to day 28 after the VARTD, which defined as a reduction of 75% or greater in size of necrotic collection (in any axis) on CT and clinical resolution of sepsis or organ dysfunction within the first 4 postoperative weeks. Baseline CECT-derived parameters were measured before the VARTD. Repeat CECT scan was performed routinely for a period of 7–14 days, or when less than 50 mL per day fluid drained was observed, or in case of clinical suspicion of enteral/pancreatic fistula or intra-abdominal bleeding. The secondary efficacy endpoint was reintervention on for an additional debridement/necrosectomy. The primary safety endpoint was a composite of major complications comprising enterocutaneous or pancreatic fistula, visceral perforation, and intra-abdominal hemorrhage that require intervention; new-onset organ failure; in-hospital death, and death within 6 months after discharge. The secondary safety endpoints included individual primary endpoint components, biliary strictures, incisional hernia, wound infections, pancreatic endocrine and exocrine insufficiency, and intensive care unit (ICU) and hospital length of stay after the VARTD. Definitions of the safety endpoints were consistent with previous reports [4, 28].

### Statistical analysis

The sample size of this study was estimated using software PASS version 15 (NCSS, LLC. Kaysville, Utah, USA). Based on published data, incidence rates of major complications/death are 40–69.5% for minimal-access surgical management in patients with necrotizing pancreatitis [4, 15–17]. On the basis of the assumption that the safety of the VARTD approach is comparable to that of the minimal-access approaches reported in previous studies, at one-sided 95% confidence interval and 85% statistical power of the study, an estimated sample size of 19 was determined. A total of 21 eligible participants are, therefore, planned, assuming a 10% dropout rate in the study.

Since this was an exploratory study, we used descriptive statistics to summarize the findings. All analyses were followed an intention-to-treat principle. Continuous variables were described using mean ± standard deviation (SD), median and interquartile range (IQR), and range. Categorical variables were reported as numbers and proportions. The statistical analyses were carried out using SPSS software version 20 (IBM Inc., Chicago, Illinois, USA).

## Results

### Demographics and baseline characteristics of patients

Between July 18, 2018, and November 12, 2020, a total of 95 NP patients admitted to our high-volume pancreatic center were screened, 66 of whom developed WON; of these, except for 1 patient who was eligible but declined participation, 21 iWONde patients (mean age 42.9 years [SD, 11.7]; 10 [48%] women) were finally enrolled and underwent the VARTD (Fig. 1).

The demographics and baseline characteristics of the study participants are presented in Table 1 (individual participant data are provided in Additional file 1: Table S1). The majority of patients (17, 81%) had a modified CT severity index (MCTSI) score higher than 8. More than half of the participants (13, 62%) were reported to have areas of non-enhancement of the pancreatic parenchyma > 50% on CECT scan. Most participants (17, 81%) reported at least one ICU stay before receiving the VARTD. The sizes and margins of the necrotic collection are shown in Table 1 and Additional file 1: Table S2. All patients had necrotic collections extending to lower abdominal regions, and the majority of (17, 81%) of the participants had extensive necrosis reaching the pelvis. Mean (SD) size of necrotic collection-anteroposterior (AP) axis was 7.9 (2.6) cm; mean (SD) size of necrotic collection-transverse axis was 13.8 (5.3) cm. Percutaneous catheters were left in situ in 19 (90%) patients prior to the VARTD approach. Detailed intraoperative information appears in Additional file 1: Table S3.

**Table 1:** Patient demographics and baseline characteristics

Variable	VARTD (*n* = 21)
*Age, years*	
Mean (SD)	42.9 (11.7)
Median (IQR)	44.1 (32.3‒53.0)
Range	23.7‒61.5
Sex, *n* (%)	
Female	10 (48)
Male	11 (52)
*Cause of pancreatitis, n* (%)	
Gallstones	6 (29)
Alcohol	4 (19)
Hypertriglyceridemia	5 (24)
Idiopathic	5 (24)
Excessive eating	1 (5)
*Body mass index, kg/m*^2^†	
Mean (SD)	22.8 (3.3)
Median (IQR)	21.9 (20.3‒26.1)
Range	18.0‒29.0
*Comorbid conditions, n* (%)	
Cardiovascular disease	3 (14)
Pulmonary disease	2 (10)
Diabetes mellitus	5 (24)
Chronic renal disease	0
Smoking history, *n* (%)	9 (43)
Drinking history, *n* (%)	6 (29)
ICU/high acuity care before enrollment, *n* (%)	17 (81)
Multiple organ failure, *n* (%)	7 (33)
Single-organ failure, *n* (%)	5 (24)
*Acute physiology score*	
Mean (SD)	7.5 (3.2)
Median (IQR)	7.0 (5.0‒10.0)
Range	3.0‒16.0
*APACHE II score*	
Mean (SD)	8.7 (3.2)
Median (IQR)	8.0 (6.0‒10.5)
Range	3.0‒16.0
*Modified CT severity index, n (%)*	
6	4 (19)
8	3 (14)
10	14 (67)
*Non-enhancement of the pancreatic parenchyma, n (%)*	
< 30%	3 (14)
30%‒50%	5 (24)
> 50%	13 (62)
*Size of necrotic collection—anteroposterior axis, cm*	
Mean (SD)	7.9 (2.6)
Median (IQR)	7.3 (5.6‒10.1)
Range	4.7‒12.9
*Size of necrotic collection—transverse axis, cm*	
Mean (SD)	13.8 (5.3)
Median (IQR)	11.8 (9.8‒18.9)
Range	6.2‒22.2
Necrosis extending to the right of mesenteric vessels, *n* (%)	21 (100)
*Distance between necrosis and left paracolic gutter, cm*	
Mean (SD)	0
Median (IQR)	0
Range	0
*Distance between necrosis and right paracolic gutter, cm‡*	
Mean (SD)	5.9 (1.2)
Median (IQR)	6.4 (4.5‒6.8)
Range	4.2‒6.9
*Distance from lower edge of the necrosis to pelvis, cm§*	
Mean (SD)	2.2 (0.9)
Median (IQR)	2.0 (1.5‒3.1)
Range	1.4‒3.4
*Necrosis extending down left paracolic gutter, cm*	
Mean (SD)	16.0 (5.6)
Median (IQR)	16.9 (12.1‒19.0)
Range	7.0‒29.8
*Necrosis extending down right paracolic gutter, cm*	
Mean (SD)	8.2 (4.8)
Median (IQR)	7.2 (3.9‒13.2)
Range	2.7‒17.0
Percutaneous catheter in situ prior to VARTD, *n* (%)	19 (90)
*Time from onset of pancreatitis to surgery, days*	
Mean (SD)	64.0 (29.7)
Median (IQR)	54.0 (41.0‒82.5)
Range	30.0‒128.0
Tertiary referral, *n* (%)	8 (38)
*Length of stay prior to our tertiary center, days*	
Mean (SD)	40.0 (36.0)
Median (IQR)	31.0 (9.5‒58.5)
Range	0‒122.0

^†^Data were measured at admission in our tertiary referral center. ‡Only 6 participants were included, and 15 others (71.4%) had extensive necrosis reaching the right paracolic gutter. §Only 4 participants were included, and 17 others (81.0%) had extensive necrosis reaching the pelvis. VARTD, video-assisted retroperitoneal and/or transperitoneal debridement; ICU, intensive care unit; APACHE, Acute Physiology and Chronic Health Evaluation

### Efficacy outcomes

The primary efficacy endpoint was achieved by most of the iWONde participants (14/21, 67%) who enrolled in the present study and underwent the VARTD approach (Table 2 and Additional file 1: Table S4). None of the study participants required repeated interventions for additional drainage or debridement. Taken together, these satisfactory efficacy outcomes in the present study suggest that the combined VARTD technique and continuous postoperative lavage are reasonably sufficient for evacuating extensive WON and achieving good clinical results.

**Table 2:** Primary and secondary efficacy endpoints

Efficacy endpoints	VARTD (*n* = 21)
*Primary efficacy endpoints*	
Reduction in size of necrotic collection after VARTD, *n* (%)	
≥ 75% reduction in any axis	14 (67)
≥ 75% reduction in transverse axis	13 (62)
≥ 50% reduction in transverse axis	19 (90)
≥ 75% reduction in AP axis	9 (43)
≥ 50% reduction in AP axis	16 (76)
Clinical resolution of sepsis or organ dysfunction, n (%)	21 (100)
Secondary efficacy endpoint	
Repeated interventions for additional drainage/debridement, *n* (%)	0

VARTD, video-assisted retroperitoneal and/or transperitoneal debridement; AP, anteroposterior

### Safety outcomes

Results for the primary and secondary safety endpoints are summarized in Table 3. The primary composite safety endpoint occurred in six participants (29%). The composite rate of reoperation was 24%(5/21), but none for debridement. The most common major complication was enterocutaneous fistula which were six cases (29%), but only three of whom were reoperated on for an ileostomy while one case was successfully treated with endoscopic clip closure, and the rest did not require intervention (Additional file 1: Table S5). The other major complications were as follows: three patient (14%) suffered intra-abdominal hemorrhage, two of whom underwent reoperation for hemostasis; one case (5%) experienced a gallbladder-abscess cavity fistula requiring a reoperation for fistula excision and repair. In this study, we observed no visceral perforation event. There was one in-hospital death (5%) in a patient who experienced repeated intra-abdominal bleeding. One case (5%) developed new-onset multiple organ failure involving heart and kidney. One case (5%) developed pulmonary embolism (unrelated to the treatment) and expired three months after discharge. The secondary safety endpoint of new-onset diabetes was occurred in three patients (14%); three patients (14%) developed pancreatic insufficiency; biliary stricture occurred in two cases (10%). All participants developed wound infection, mainly occurred in the flank incisions at the “port-site” of postoperative lavage tubes. Only five patients (24%) transferred to ICU after surgery; of these, one case (5%) was attributed to the new-onset cardiac and renal failure, and the others failed early postoperative extubation and required a transient critical care. Median length of postoperative ICU stay was 3 days (IQR 1–4).

**Table 3:** Primary and secondary safety endpoints

Safety endpoints	VARTD (*n* = 21)
Primary composite endpoint, *n* (%)	6 (29)
*Primary endpoint components, n (%)*	
Death	2 (10)
New-onset organ failure	1 (5)
Enterocutaneous fistula†	4 (19)
Pancreatic fistula†	0
Other fistulae‡	1 (5)
Intra-abdominal bleeding†	2 (10)
Visceral perforation†	0
*Secondary endpoints*	
Endocrine and exocrine function, *n* (%)	
New-onset diabetes	3 (14)
New diagnosis of pancreatic insufficiency	3 (14)
Postoperative ICU admission, *n* (%)	5 (24)
Length of postoperative ICU stay, days	
Mean (SD)	2.6 (1.7)
Median (IQR)	3.0 (1.0‒4.0)
Range	1.0‒5.0
Biliary strictures, *n* (%)	2 (10)
Incisional hernia, *n* (%)	0
Wound infections, *n* (%)	21 (100)
Length of hospital stay after VARTD, days	
Mean (SD)	51.8 (33.1)
Median (IQR)	40.0 (31.0‒63.0)
Range	24.0‒139.0
Length of stay at our tertiary center, days	
Mean (SD)	74.7 (41.2)
Median (IQR)	68.0 (41.0‒89.5)
Range	32.0‒175.0
Length of stay after the onset of pancreatitis, days	
Mean (SD)	114.9 (42.1)
Median (IQR)	115.0 (81.0‒135.5)
Range	63.0‒236.0

^†^Only the complications requiring additional intervention were included. ‡Other types of fistulae requiring additional intervention: gallbladder-abscess cavity fistula (*n* = 1); gastric antral fistula (*n* = 0). VARTD, video-assisted retroperitoneal and/or transperitoneal debridement; ICU, intensive care unit

## Discussion

Currently, a step-up approach applying PCD and other minimal-access interventions has been proven to have more favorable outcomes for the management of WON [2, 3, 7, 9, 14–18]. However, according to the present expert consensus and updated guideline, open surgery is still an appropriate option for the infected WON with deep extension [2, 3, 10, 12, 13, 24–27]. In this study, we show that a minimal-access approach followed by continuous postoperative lavage, as an optimization method, could achieve the goal, as much as possible, of debridement of the extensive infected necrotic collection without additional interventions and with reasonably low incidence rates of major complications/death.

Although several therapeutic schemes have emerged and significant progress towards reducing mortality and the risk of medical complications has been made in the treatment for NP during the last decade, it is clear that sufficient drainage and/or debridement remain the most important component in the management of WON, a delayed but life-threatening NP complication [1–3, 5, 9]. Importantly, there is a major limitation regarding the use of one stand-alone minimally invasive technique within the step-up approach, regardless of ETD/ETN, STE or VARD, in the treatment of infected WON with deep extension that is spread throughout the abdomen. The main reason is that the access route for each intervention type fails to reach necrotic collections located in not only the pancreatic parenchymal area but also multiple of the peripheral zones including retromesenteric plane and/or either paracolic gutter, leading to insufficiently evacuation of semi-solid necrotic debris.

The VARTD technique allows for good efficacy while evacuating the necrotic collections that located both centrally and diffusely throughout the abdomen. The continuous postoperative lavage also provides advantage in constant evacuations of necrotic debris, inflammatory exudate, vasoactive and toxic products, active enzymes, and bacteria and the toxins thereof [5, 29]. In this study, large-bore drains were crosswire placed in the necrotic cavity as well as alongside the lateral retroperitoneal access routes so as to easily flushed out the remaining necrotic debris with an elevation effect from high to low position. Most participants receiving the VARTD in our study showed effective evacuation of necrotic collections within 4 weeks postoperatively. Notably, none of the participants required additional interventions to remove the rest of the necrotic debris. It should be noted that the NP patients participated in the present study had a larger necrosis burden and a more serious condition when compared with those of the participants in other previous studies. Obviously, the rate of reinterventions after the VARTD was much lower than that of the use of an endoscopic or a minimal-access surgical approach alone [19, 22, 23, 25, 28]. The reintervention rates following the VARD or ETD/ETN reported in a previous study were 37.5% and 44.1%, respectively [28]. Furthermore, some patients undergoing the VARD or ETD/ETN even required more than three necrosectomy or endoscopic drainage procedures [25, 28]. However, there is no denying the fact that combination of endoscopic approaches via multiple transmural sites (multigate technique) and PCD or VARD have a potential to be another good choice for the patients who had developed iWONde [1–3, 21, 30]. Whether the VARTD is superior to the combined endoscopic transgastric drainage/necrosectomy and VARD approaches cannot be clearly concluded, future research may come to give a clear answer. As well, comparison of the cost efficiency between the VARTD and other approaches is worthy of consideration in future prospective studies.

Results reported here also indicate an acceptable safety profile of the VARTD approach. The rates of postoperative complication-morbidity and mortality of the VARTD were substantially lower when compared with those of open surgical debridement as previously reported [9, 11, 17, 25]. In the present study, only one participant (5%) developed new-onset multiple organ failure. Such incident rate is much lower than that of open surgery (approximately 40%) [17]. More importantly, the mortality rate (10%) attributable to the VARTD technique was comparable to that in Bang et al. (endoscopic 8.8% or VARD 6.3%) [28] and van Brunschot et al. (endoscopic 18% or VARD 13%) as well [4]. Furthermore, postoperative ICU length of stay for the study participant receiving VARTD (median, 3 days) was considerably less than that for patients who had undergone open surgery (19 days) [18]. In addition, of the 21 participants enrolled in this study, only 5 patients (24%) required reoperation for addressing the postoperative complications such as enteral/pancreatic fistula and intra-abdominal bleeding. Thus, the VARTD offers a reasonably safe strategy for evacuation of the iWONde.

Percutaneous catheter drainage or endoscopically transluminal drainage for necrotic collection is preferably postponed, usually > 4 weeks after the disease onset, waiting until necrosis has been encapsulated [31]. Furthermore, though clinical condition is sometimes changed rapidly owing to pathophysiological events initiated by infection at early phase of necrotizing pancreatitis, a recent POINTER trial conducted by Dutch Pancreatitis Study Group found that immediate catheter drainage did not provide more benefits for the NP patients [32]. In the present study, we performed the VARTD while infected WON was developed spreading over the abdomen. However, we were wondering whether early application of the VARTD prior to the current standard “step-up” approach in selected NP patients in particular circumstances could prevent further clinical worsening. It is, therefore, worthwhile to consider the timing for performance of the VARTD in future research.

This was a proof-of-concept study and has some limitations. The small sample size and lack of the risk factor-matched control groups do not allow us to make conclusions about the real safety of the VARTD. Such may also lead to an overestimation of effectiveness of this technique in the face of improving patient outcomes, thus its efficacy should be interpreted in mind. Furthermore, a potential discrepancy between the participants initially treated at primary hospitals and at tertiary referral centers regarding patterns of clinical management prior to receiving the VARTD was not resolved, advertising the potential for an indispensable selection bias. In addition, there remain limited knowledge regarding infected WON with deep extension, and there is no consensus on standard definition for its clinical improvement, thus our a priori definition may result in design bias. This may lead to a reduction in the reintervention rate after the VARTD as compared with other minimal-access techniques as reported previously.

## Conclusions

In this study, the VARTD was performed as safely as the currently preferred minimal-access surgical approaches for the treatment of infected WON; the VARTD combined with continuous postoperative lavage showed a good efficacy for evacuating necrotic debris while avoiding additional interventions; thus, this approach may be an option for selected patients especially the ones developed the iWONde after failure of the “step-up” approach. To test these findings further, a large-scale, randomized trial involving multiple institutions and comparing the effects of the VARTD with other minimal-access techniques on outcomes of patients who had developed iWONde is warranted.

## Supplementary Information

**Additional file 1**. The following are available as supplementary data in the Additional files: **Table S1** Individual participant data for demographics and baseline characteristics. **Table S2** Measurements of CT-derived variables of necrosis at an individual level. **Table S3** Intraoperative data. **Table S4** Efficacy endpoints at an individual level. **Table S5** Safety endpoints at an individual level.

## Data Availability

Deidentified individual participant data used and/or analyzed during the current study are available in Additional file 1: Tables S1–S5.

## Abbreviations

ANCs: Acute necrotic collections
AP: Anteroposterior
CECT: Contrast-enhanced computed tomography
ETD/ETN: Endoscopic transluminal debridement/necrosectomy
ICU: Intensive care unit
IQR: Interquartile range
iWON: Infected walled-off pancreatic necrosis
iWONde: IWON with deep extension
VARTD: Video-assisted retroperitoneal and/or transperitoneal debridement
NP: Necrotizing pancreatitis
PCD: Percutaneous catheter drainage
SD: Standard deviation
STE: Sinus tract endoscopy
VARD: Video-assisted retroperitoneal debridement
